# Ovarian Fetiform Teratoma in a 17-Year-Old Adolescent Girl

**DOI:** 10.7759/cureus.15644

**Published:** 2021-06-14

**Authors:** Khalid A Al Wadi, Hanan A Mal, Muhammad Amin Ur Rahman, Mohammed Abuzaid, Ahmed Abu-Zaid

**Affiliations:** 1 Department of Obstetrics and Gynecology, King Fahad Medical City, Riyadh, SAU; 2 Department of Pathology, King Fahad Medical City, Riyadh, SAU; 3 College of Graduate Health Sciences, The University of Tennessee Health Science Center, Memphis, USA

**Keywords:** fetiform teratoma, homunculus, fetus-in-fetu, ovarian neoplasms, ectopic pregnancy

## Abstract

Fetiform teratoma, also recognized as a homunculus, is a largely uncommon form of mature cystic teratoma. Here, we present the case of a 17-year-old single female who presented to the emergency department complaining of abdominal distension and pain for four months. Abdominal examination revealed a left-sided mass. Magnetic resonance imaging showed a multi-loculated and multi-septated left cystic ovarian mass, suspicious for a teratoma. The patient underwent laparoscopy and a left cystectomy was performed. The final histopathologic diagnosis was consistent with fetiform teratoma. Although extremely rare, ovarian fetiform teratoma should be considered in the differential diagnosis of women presenting with an abdominopelvic mass. It should be discerned from fetus-in-fetu and ectopic pregnancy. Careful clinical presentation, laboratory testing for beta-human chorionic gonadotropin, histopathologic examination, and cytogenetic analysis can greatly aid in pinpointing the diagnosis. Overall, fetiform teratoma carries a favorable prognosis; however, follow-up surveillance is advised to monitor for uncommon occasions of tumor persistence or relapse.

## Introduction

Fetiform teratoma, also recognized as a homunculus, is a largely uncommon form of mature cystic teratoma. It is characterized by the presence of a highly differentiated mass that closely parallels a fetus-like organization [1]. Although ovaries are the most frequently implicated sites, several unusual anatomical regions have also been reported, such as retroperitoneum [2], cecum [3], and sacrococcyx [4]. It is exceedingly uncommon with very limited cases documented to date in the English-language literature. It should be carefully discriminated from its closely related differential diagnoses of fetus-in-fetu and ectopic pregnancy [5]. Here, we report a case of ovarian fetiform teratoma in a 17-year-old adolescent girl.

## Case presentation

A 17-year-old single female presented to the emergency department complaining of abdominal distension and pain for four months. Past medical, surgical, and gynecologic histories were unremarkable. Moreover, prenatal history was not significant for any concerns.

Physical examination showed normal vital signs. Secondary sexual characteristics were well developed. On abdominal examination, a well-defined, smooth, firm, and mobile mass was felt on the left side extending to the midline and four fingers above the umbilicus. There was no tenderness or rigidity. Laboratory testing for tumor markers revealed normal alpha-fetoprotein, normal beta-human chorionic gonadotropin (beta-HCG), normal lactate dehydrogenase, slightly increased cancer antigen 125 (48.8 U/mL; normal value <46 U/mL), and moderately increased carcinoembryonic antigen (9.3 ng/mL; normal value <3 ng/mL).

**Figure 1:**
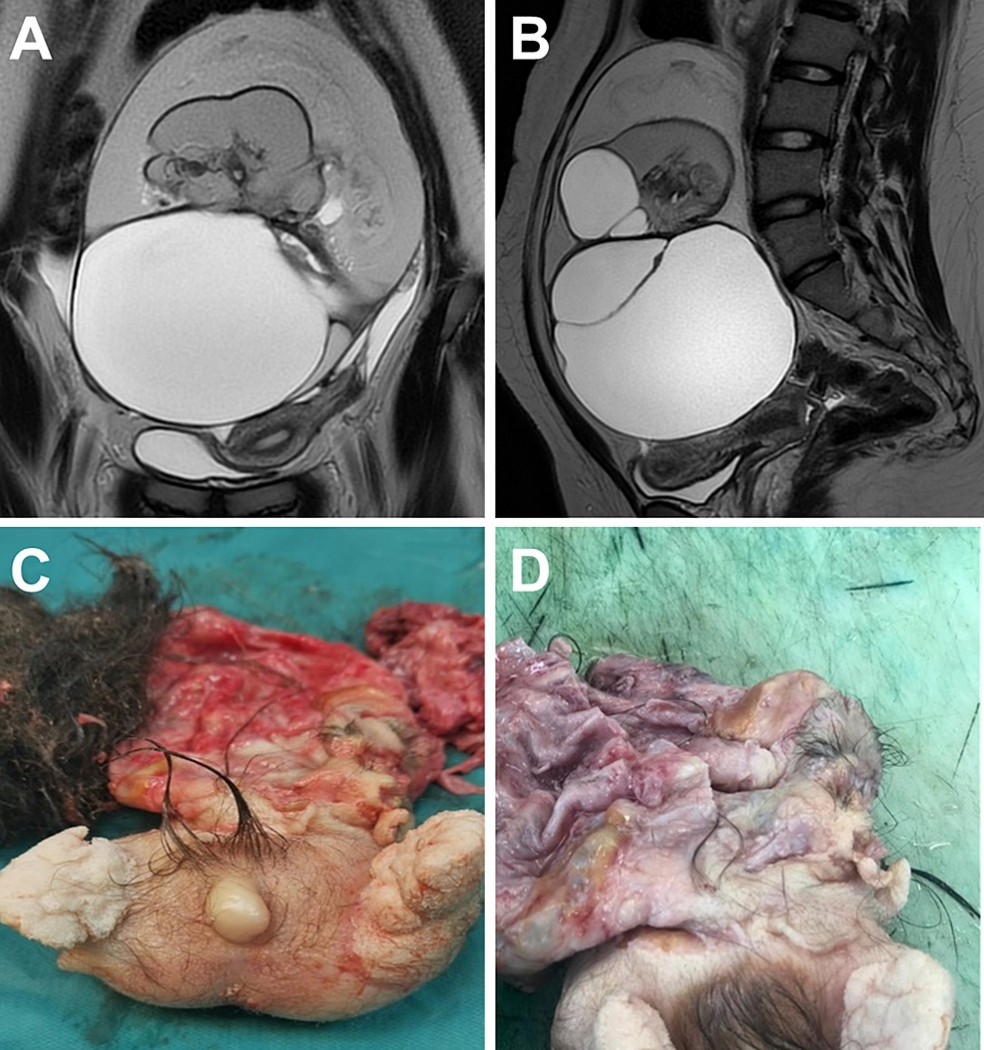
(A) Coronal and (B) sagittal magnetic resonance images showing a large left ovarian mass. (C) Gross morphology of the tumor showing two rudimentary limbs and a penis-like structure. (D) The tumor consisted of cystic (upper) and solid (lower) components.

Ultrasonography revealed a pelvic cystic mass measuring 15 × 11 cm. There was no pelvic free fluid. Both ovaries were not visualized. Magnetic resonance imaging (MRI) showed a multi-loculated and multi-septated left cystic ovarian mass (Figures 1A, 1B). It also showed areas of extracellular and intracellular fatty components. There were multiple areas of high T2-weighted images representing fluid components. There was no suspicious enhancing soft tissue component. Overall, the mass measured 20 × 16 × 10 cm. It was causing a mass effect on the uterus and ureters. The uterus had normal zonal demarcation without focal lesion, and the right ovary was unremarkable. There were no suspicious pelvic lymphadenopathies or obvious signs of peritoneal disease. A differential radiological diagnosis of teratoma was suspected.

In view of possible underlying malignancy, the patient underwent laparoscopy. The left ovarian cystic mass was identified and cystectomy was performed. The left ovarian mass was consistent with a dermoid cyst and grossly looked like a malformed fetus. The remaining anatomy was within normal limits.

Macroscopically, the left ovarian cystic mass weighed 294 g and measured 12 × 9 × 5.6 cm. After opening the mass, it appeared heterogeneous with multi-locular cystic and solid areas (Figures 1C, 1D). The solid component appeared to be a malformed fetus with a deformed “head” region consisting of ill-formed flat bones attached to the cystic component and a “trunk” region with well-formed skin. The trunk also showed three limb-like protrusions (rudimentary extremities), one smaller upper and two larger lower extremities, and a small penis-like protrusion between the two lower limb protrusions. Hair was distributed throughout the mass, but more so in the “head” region. The cut surfaces of the solid component showed well-formed skin and subcutaneous fat (the bulk of the solid mass). The limb-like protrusions contained bone and cartilage in the center.

**Figure 2:**
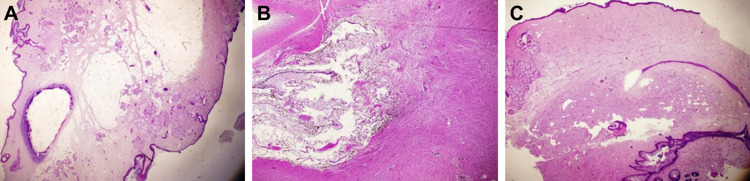
(A) Microscopic hematoxylin and eosin stain of the limb-like protrusion showing skin on both sides with central cartilage and scanty bone. (B) Microscopic hematoxylin and eosin stain showing neural tissue (right and lower) with pigmented retinal tissue (left). (C) Microscopic hematoxylin and eosin stain from the external genitalia (penis-like structure) showing urethra (lower right) flanked by erectile tissue.

Microscopically, the mass revealed mature anlage mainly from the embryonic ectoderm and mesoderm consisting of skin, subcutaneous fat, cartilage, bone, and abundant mature neural tissue including pigmented retinal tissue (Figures 2A, 2B). The penis-like protrusion revealed ill-formed male genital structures consisting of prostatic tissue, urethral structure (lined by urothelium) surrounded by erectile tissue (corpus cavernosum). Interestingly, the urethral structure was seen extending from the prostatic tissue and distally flanked by the erectile tissue reminiscent of the normal male genital system (Figure 2C). No immature elements or malignancy were seen.

The final histopathologic diagnosis was consistent with fetiform teratoma. After surgery, the patient recovered without complications and was discharged from the hospital after two days. At the 13-month follow-up, the patient was doing well without proof of recurrence.

## Discussion

Mature ovarian teratomas, also known as dermoid cysts, are common and account for nearly 20-40% of all ovarian neoplasms [6]. They are composed of mature tissues arising from one or more germ layers, namely, the endoderm, mesoderm, and ectoderm [7]. These mature tissues, such as the epidermis, hair, and teeth, are oriented in a disorganized manner inside the cysts. However, despite being exceedingly uncommon, these mature cystic teratomas can assume a high extent of organized differentiation and macroscopically bear a close resemblance to a distorted fetus, a condition known as fetiform teratoma or homunculus [1]. The incidence of fetiform teratoma is exceptionally rare with a very limited number of reported cases worldwide.

Fetiform teratoma should be discerned from fetus-in-fetu and ectopic pregnancy. At present, it seems that fetiform teratoma and fetus-in-fetu are not two discrete conditions, but rather two manifestations of the same pathologic entity at dissimilar phases of maturation [8]. Accumulating evidence has illuminated several major findings to discriminate between fetiform teratoma and fetus-in-fetu [1,8-10]. Clinically, fetiform teratoma most often arises in ovaries of reproductive-aged women, whereas fetus-in-fetu is commonly localized retroperitoneally in infants. From a gender perspective, cases of fetus-in-fetu and fetiform teratoma are more commonly observed in males and females, respectively [8]. Structurally, fetiform teratoma does not contain a vertebral column and internal organs, whereas fetus-in-fetu contains a vertebral column with internal organs and limb buds around the central axis. Molecularly, fetiform teratoma is homozygous relative to its host heterozygosity, whereas fetus-in-fetu is genetically alike to its host. We could not carry out cytogenetic evaluation for zygosity as the tissues were fixed with formaldehyde. From a prognostic point of view, fetus-in-fetu is almost always benign, whereas fetiform teratoma can exhibit malignant transformation [10]. Nonetheless, collectively, our case presentation largely fits the description of fetiform teratoma, rather than fetus-in-fetu.

Fetiform teratoma arising from ovaries can sometimes be confused with ectopic pregnancy. Histologically, fetiform teratoma consists of mature tissue without trophoblastic/chorionic tissue [1]. The presence of the latter tissue is highly suggestive of potential ectopic pregnancy. Moreover, increased beta-HCG levels along with pathologic identification of trophoblastic/chorionic tissue can fundamentally hint toward ectopic pregnancy diagnosis [1]. Our case presentation demonstrated negative beta-HCG levels and no histopathologic evidence of trophoblastic/chorionic tissue, thus fetiform teratoma diagnosis was substantiated.

Overall, the prognosis of fetiform teratoma is favorable [11]. Despite largely benign, up to 2% of mature cystic teratomas are malignant, and close to 10% of them recur post-resection [6]. Thus, complete resection of the tumor is advised along with follow-up surveillance comprising laboratory and imaging testing to scrutinize for tumor persistence or relapse [6].

## Conclusions

We reported a rare case of ovarian fetiform teratoma in a 17-year-old adolescent girl. This is the first report from Saudi Arabia. Although exceedingly uncommon, ovarian fetiform teratoma should be considered in the differential diagnosis of women presenting with an abdominopelvic mass. Fetiform teratoma should be discerned from fetus-in-fetu and ectopic pregnancy. Careful clinical presentation, laboratory testing for beta-HCG, histopathologic examination, and cytogenetic analysis can greatly aid in pinpointing the diagnosis. Overall, fetiform teratoma carries a favorable prognosis; however, follow-up surveillance is advised to monitor for uncommon occasions of tumor persistence or relapse.
